# A Method to Address the Impact of Incident Conditions on the Spectral Reconstruction of the Talbot Wavemeter

**DOI:** 10.3390/s25051609

**Published:** 2025-03-06

**Authors:** Yiming Wang, Yu Huang, Xiaohu Yang, Zhanfeng Li, Yue Li

**Affiliations:** 1 Changchun Institute of Optics, Fine Mechanics and Physics, Chinese Academy of Sciences, Changchun 130033, China; wangyiming216@mails.ucas.ac.cn (Y.W.); yangxiaohu861106@163.com (X.Y.); lizhanfeng115@163.com (Z.L.); liyue@ciomp.ac.cn (Y.L.); 2 University of Chinese Academy of Sciences, Beijing 100049, China

**Keywords:** talbot wavemeter, incident conditions, alignment error, Moiré fringe

## Abstract

The Talbot wavemeter has attracted widespread attention from researchers in recent years due to its advantages of miniaturization and low cost. However, the impact of varying incident conditions caused by factors such as alignment has remained a challenge for spectral retrieval. This paper first derives the influence of different incident conditions on the interference pattern based on Fresnel diffraction and verifies the derivation through simulations. We propose a method to address the impact of incident conditions on the interference pattern. By adding a grating with a different periodicity in front of the detector, Moiré fringes are generated in the periodicity dimension, increasing the fringe period and thus enlarging the tolerance for angular misalignment. Finally, we constructed a Talbot wavemeter based on a double-grating structure, achieving a spectral resolution of 9 nm at 360 nm. This method provides a reference for the future development of a high-precision, high-resolution Talbot wavemeter.

## 1. Introduction

The precise measurement of optical wavelengths plays a crucial role in various fields such as analytical chemistry, biosensing, material analysis [1], and optical communications [2,3]. Optical wavelength measurement instruments, known as wavemeters, are traditionally based on interferometric measurements, including Michelson, Fabry–Perot, and Fizeau types. The Michelson wavemeter offers high measurement accuracy [4,5]; it collects interference signals by moving a reference mirror and then obtains wavelength information through the Fourier transform. However, it contains moving parts, which impose high stability requirements. The Fabry–Perot wavemeter measures the wavelength of the laser under test by utilizing multi-beam interference generated when the light beam passes through two glass plates with high reflectivity and fixed spacing. Although it provides relatively high measurement accuracy, it requires an integrated reference laser [6]. The Fizeau wavemeter, on the other hand, does not require an integrated reference light source and has no moving parts, but its measurement accuracy is relatively lower [7]. Moreover, all interferometric wavelength measurement systems are highly sensitive to temperature variations and require strict temperature control. In recent years, with the development of optical technology and deep learning, various new wavelength detection methods have emerged. For example, the wavemeter based on Rayleigh speckle [8] analyzes the random sawtooth pattern generated when Rayleigh backscattered light in a single-mode optical fiber interferes, thus obtaining the wavelength information of the incident light [9]. Another example is the Talbot effect wavemeter, which can detect the wavelength of incident light using only a plane transmission grating and a detector [10]. It offers low cost and miniaturization, which has attracted widespread attention from researchers in recent years.

The Talbot effect refers to the phenomenon where incident light, when illuminated on a periodic structure, can self-image at a specific distance behind the structure. Due to this phenomenon, the Talbot effect has been increasingly applied in various optical measurements in recent years. For example, Song et. al. introduced a novel displacement measurement method that utilizes the missing-order Talbot effect and a high-precision two-dimensional angular sensor [11,12]. The measurement dynamic range can be adjusted by altering the grating period and the wavelength of the incident light. The angular sensor achieves a detection resolution of 0.4 arcseconds and a dynamic range exceeding 1400 arcseconds. Yang et. al. introduced an ultracompact angular displacement sensor based on the Talbot effect of optical microgratings [13]. A sensitivity of 0.19 mV/arcsec was experimentally obtained, leading to a relative sensitivity of 0.27%/arcsec within a linear range of ±396 arcsec with the 2 μm period optical gratings. Since the Talbot distance is related to the wavelength of the incident light, an inclined planar detector is placed behind the grating to capture the Talbot self-imaging pattern [14]. By performing a Fourier transform on the captured image, the wavelength information of the incident light can be obtained. Helen et al. proposed a Talbot spectrometer that utilizes a grating with a 5 µm period to generate Talbot fringes [10]. A lens was used to image the interference pattern onto the detector surface, enabling wavelength measurement of green (543 nm) and red (633 nm) lasers, achieving a spectral resolution of 42 nm. Subsequently, Erika et al. introduced a lensless spectrometer based on the Talbot effect, which achieved both miniaturization (<0.6 cm^3^) and high resolution (<1 nm). They also analyzed the linear relationship between the measured wavelength and the laser wavelength [14]. Since the Talbot effect requires highly coherent light incident on a periodic structure, researchers have increasingly applied this technique to wavemeters rather than spectrometers as technology has advanced. Han et al. combined signal processing with the Talbot wavemeter, achieving a single estimation uncertainty of less than 10 pm under the 3-δ criterion [15]. Although the Talbot wavemeter has the advantages of miniaturization and low cost, and its performance has been relatively developed, it still faces a significant issue: the impact of incident light conditions on the Talbot wavemeter. It is known that the Talbot effect occurs when parallel light illuminates a periodic structure. However, during beam expansion and collimation of the laser beam, low collimation may cause the incident light to shift from a plane wave to a spherical wave. Additionally, alignment errors may result in an angle between the incident light and the grating. Therefore, analyzing and addressing the influence of incident conditions on the Talbot wavemeter is of paramount importance.

This paper first derives the precise wave function of the Talbot effect under different incident light conditions based on Fresnel diffraction theory, analyzing the impact of various incident light conditions on the self-imaging of the Talbot effect. The theoretical conclusions are then verified through simulations, and the influence of different incident conditions on spectral reconstruction is examined. A method is proposed to improve alignment tolerance by adding an additional grating. Finally, a Talbot wavemeter is constructed based on this method, achieving a spectral resolution of 9 nm at 360 nm.

## 2. Theory

### 2.1. Parallel Light Normal Incident

**Figure 1:**
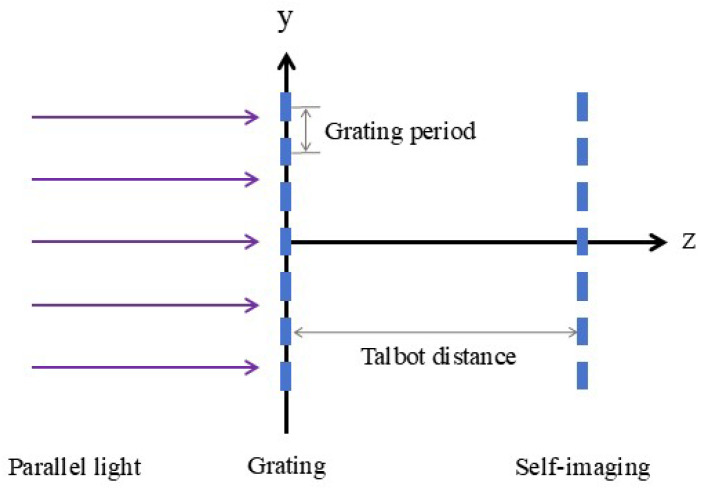
Diagram of Talbot effect when parallel light is normally incident on a grating.

As shown in Figure 1, when a parallel light is incident perpendicularly on the grating, a self-image of the grating is formed at the Talbot distance behind the grating. We proceed with a theoretical derivation. Let the amplitude of the plane wave be *A*. The wave function of the plane wave on the front surface of the grating can be expressed as

(1) $$E_{1}=A$$

The transmission function of the grating can be expressed as

(2) $$g\left(y_{0}\right)=\sum_{-\infty }^{\infty }C_{n}e^{i2\pi \frac{n}{d}y_{0}}$$

where *C_n_* represents the Fourier transform component of the grating transmission function [16], *n* represents the number of grooves on the grating, and *d* represents the grating period, then the wave function of the light behind the grating can be expressed as

(3) $$E_{2}=E\cdot g\left(y_{0}\right)=A\sum_{-\infty }^{\infty }C_{n}e^{i2\pi \frac{n}{d}y_{0}}$$

Therefore, in the near field region of the Fresnel approximation [17], the exact wave function propagating to the *z* position can be expressed as

(4) $$E\left(y,z\right)=\frac{e^{ikz}}{i\lambda z}e^{i\frac{k}{2z}y^{2}}\int A\sum_{-\infty }^{\infty }C_{n}e^{i2\pi \frac{n}{d}y_{0}}e^{i\frac{k}{2z}y_{0}^{2}}e^{-i2\pi \frac{y}{\lambda z}y_{0}}dy_{0}$$

where *k =* 2*π*/*λ*, and *λ* represents the wavelength of the incident light. In this case, we make the transfer function of the system in Fresnel diffraction *h*(*y*_0_, *z*); then, we have

(5) $$h\left(y_{0},z\right)=\frac{e^{ikz}}{i\lambda z}e^{i\frac{k}{2z}y_{0}^{2}}$$

Then, we obtain

(6) $$\begin{array}{l} E\left(y,z\right)=Ae^{i\frac{k}{2z}y^{2}}FFT\left\{g\left(y_{0}\right)\cdot h\left(y_{0}\right)\right\}\\ \begin{array}{ccc} & & = \end{array}Ae^{i\frac{k}{2z}y^{2}}FFT\left\{g\left(y_{0}\right)\right\}⊗FFT\left\{h\left(y_{0}\right)\right\}\\ \begin{array}{ccc} & & = \end{array}Ae^{i\frac{k}{2z}y^{2}}\sum_{-\infty }^{\infty }C_{n}\delta \left(f_{y}-\frac{n}{d}\right)⊗e^{-ikz}e^{-i\lambda z\pi f_{y}^{2}}\\ \begin{array}{ccc} & & = \end{array}Ae^{i\frac{k}{2z}y^{2}}\sum_{-\infty }^{\infty }C_{n}e^{-ikz}e^{-i\lambda z\pi {\left(f_{y}-\frac{n}{d}\right)}^{2}}\\ \begin{array}{ccc} & & = \end{array}Ae^{-ikz}\sum_{-\infty }^{\infty }C_{n}e^{i2\pi \frac{n}{d}y}e^{-i\lambda z\pi \frac{n^{2}}{d^{2}}} \end{array}$$

where *f_y_* = *y*/*λz*, ⊗ denotes the convolution operator, and *δ* function represents the Fourier transform of the grating transmission function. From this equation, it can be seen that when *z* = 2*Nd*^2^/*λ*, the last term in the expression equals 1, where *N* is a positive integer. Ignoring the constant phase factor, the wave function becomes identical to the transmission function of the grating, thus achieving self-imaging. This distance is referred to as the Talbot distance [18].

### 2.2. Parallel Light Oblique Incident

**Figure 2:**
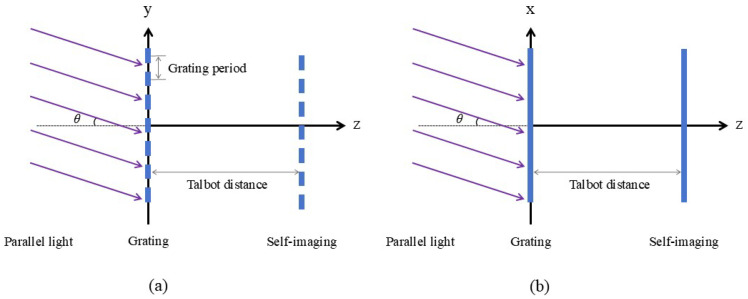
Diagram of Talbot effect when parallel light is obliquely incident (**a**) perpendicular to the grating grooves and (**b**) along the direction of the grating grooves.

Next, we perform a theoretical derivation of the Talbot effect under the condition of oblique incidence of parallel light, using a method similar to the previous section, which is based on Fresnel diffraction. First, the oblique incidence of parallel light can be divided into two directions: one is along the direction of the grating lines, and the other is along the direction perpendicular to the grating lines, as shown in Figure 2. We will first derive the case where the light is tilted in the direction perpendicular to the grating lines.

When the incident light angle is *θ*, the wave function of the incident light in front of the grating can be expressed as

(7) $$E_{1}=Ae^{i\left(k\sin\theta y_{0}+k\cos\theta z_{0}\right)}$$

Therefore, similar to the previous section, in the near-field region under the Fresnel approximation, the exact wave function at the position *z* can be expressed as

(8) $$\begin{array}{l} E\left(y,z\right)=\frac{e^{ikz}}{i\lambda z}e^{i\frac{k}{2z}y^{2}}\int Ae^{i\left(k\sin\theta y_{0}+k\cos\theta z_{0}\right)}\sum_{-\infty }^{\infty }C_{n}e^{i2\pi \frac{n}{d}y_{0}}e^{i\frac{k}{2z}y_{0}^{2}}e^{-i2\pi \frac{y}{\lambda z}y_{0}}dy_{0}\\ \begin{array}{ccc} & & = \end{array}A\frac{e^{ikz}}{i\lambda z}e^{ik\left(\sin\theta y+\cos\theta z\right)}e^{-i\pi z\frac{\sin^{2}\theta }{\lambda }}\sum_{-\infty }^{\infty }C_{n}e^{\left[i2\pi \frac{n}{d}\left(y-z\sin\theta \right)\right]}e^{-i\lambda z\pi \frac{n^{2}}{d^{2}}} \end{array}$$

From the above equation, it can be seen that when *z* = 2*Nd*^2^/*λ*, ignoring the constant phase factor, self-imaging is achieved. However, at this point, the position of the grating self-image is shifted by *z*sin*θ* along the direction perpendicular to the grating lines. Therefore, when we capture the Talbot fringes in the z-direction, the fringes will be tilted, which can lead to errors in spectral reconstruction.

Next, we derive the case where the incident light is tilted along the direction of the grating lines. When the incident light angle is *θ*, the wave function of the incident light in front of the grating can be expressed as

(9) $$E_{1}=Ae^{i\left(k\sin\theta x_{0}+k\cos\theta z_{0}\right)}$$

Since the transmission function of the grating is a function of *y*, the case where the incident light is tilted along the direction of the grating lines is similar to the case of perpendicular incidence, except for a different constant phase factor. Therefore, self-imaging is still achieved when *z* = 2*Nd*^2^/*λ*.

### 2.3. Spherical Light Normal Incident

**Figure 3:**
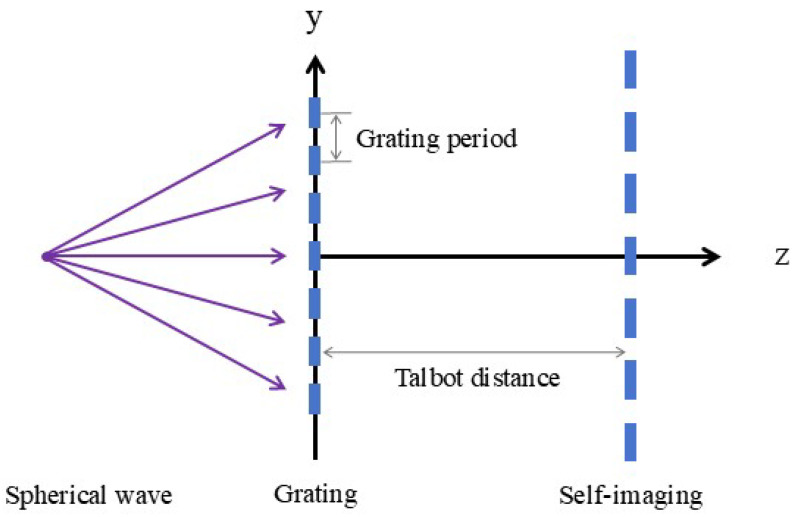
Diagram of Talbot effect when spherical light is normally incident on a grating.

Finally, we perform a theoretical derivation of the Talbot effect under the condition of normal incidence of a spherical wave emitted by a point light source. As shown in Figure 3, suppose the vertical distance between the point light source and the grating is *z*_0_, and we only consider the y-direction. The wave function of the spherical wave when it propagates to the front surface of the grating can be expressed as

(10) $$E_{1}=\frac{A}{z_{0}}e^{ikz_{0}}e^{\frac{ik}{2z_{0}}y_{0}^{2}}$$

Therefore, similar to the previous section, in the near-field region under the Fresnel approximation, the exact wave function at the position *z* can be expressed as

(11) $$\begin{array}{l} E\left(y,z\right)=\frac{e^{ikz}}{i\lambda z}e^{i\frac{k}{2z}y^{2}}\int \frac{A}{z_{0}}e^{ikz_{0}}e^{\frac{ik}{2z_{0}}y_{0}^{2}}\sum_{-\infty }^{\infty }C_{n}e^{i2\pi \frac{n}{d}y_{0}}e^{i\frac{k}{2z}y_{0}^{2}}e^{-i2\pi \frac{y}{\lambda z}y_{0}}dy_{0}\\ \begin{array}{ccc} & & = \end{array}A\frac{e^{ik\left(z+z_{0}\right)}}{i\lambda zz_{0}}e^{\frac{ik}{2\left(z+z_{0}\right)}y^{2}}\sum_{-\infty }^{\infty }C_{n}e^{\left[i2\pi \frac{n}{d}y\frac{z_{0}}{z+z_{0}}\right]}e^{-i\lambda \pi \frac{n^{2}}{d^{2}}\frac{zz_{0}}{z+z_{0}}} \end{array}$$

From the above equation, it can be seen that when *zz*_0_ + *z*_0_ = 2*Nd*^2^/*λ*, self-imaging of the grating is achieved. At this point, the Talbot distance *z* can be expressed as

(12) $$z=\frac{2Nd^{2}z_{0}}{\lambda z_{0}-2Nd^{2}}$$

Although self-imaging of the grating is achieved, the period will be magnified. The magnification factor *M* of the grating period is given by

(13) $$M=\frac{z_{0}+z}{z_{0}}$$

It is worth noting that when *z*_0_ approaches infinity, the distance between the point light source and the grating becomes infinitely large, which is equivalent to the case of parallel light incidence. At this point,

(14) $$z=\frac{2Nd^{2}z_{0}}{\lambda z_{0}-2Nd^{2}}\approx \frac{2Nd^{2}z_{0}}{\lambda z_{0}}=\frac{2Nd^{2}}{\lambda }$$

At this point, the Talbot distance is the same as the theoretical derivation for parallel light incidence.

## 3. Simulation and Analysis

In Section 2, based on our derivations, we found that when parallel light is normally incident on the grating, self-imaging of the grating occurs at the Talbot distance. When parallel light is obliquely incident along the direction perpendicular to the grating lines, self-imaging of the grating also occurs at the Talbot distance, but the self-imaging position will shift along the direction perpendicular to the grating lines. When parallel light is obliquely incident along the direction of the grating lines, there is no effect on self-imaging. When a spherical wave is normally incident on the grating, the Talbot distance depends on the distance between the point light source and the grating, and at the Talbot distance, although self-imaging of the grating occurs, the grating period will be larger. Next, we will verify the theoretical derivations through simulation.

**Figure 4:**
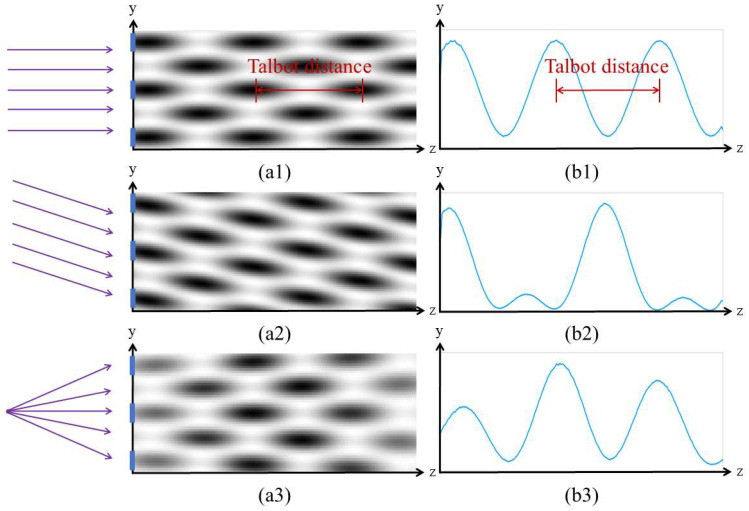
Talbot carpet and single-row Talbot fringe of the condition which (**a1**,**b1**) parallel light normal incident, (**a2**,**b2**) parallel light oblique incident, and (**a3**,**b3**) spherical light normal incident.

First, we use the optical simulation software GODAS to simulate the Talbot effect under different incidence conditions. A 360 nm laser light source is chosen for the incident light, and a sine-transmission grating with a period of 3.33 µm is used to generate the Talbot carpet. The angle of oblique incidence of the parallel light on the grating is set to 0.2°, and the distance between the point light source and the grating is 100 mm. An inclined detector is placed to collect the Talbot carpet. The pattern of the Talbot carpet and the corresponding single-row Talbot fringe are shown in Figure 4.

As shown in Figure 4(a1), when parallel light is normally incident on the grating, self-imaging and negative self-imaging alternately appear behind the grating. When the image is collected with an inclined detector, we refer to this pattern as the Talbot carpet. By selecting one row, the periodic variation curve can be observed, as shown in Figure 4(b1). Through Fourier transform, we can obtain the wavelength information of the incident light. From Figure 4(a2), it can be seen that when parallel light is obliquely incident on the grating, the Talbot carpet becomes tilted. This is due to the self-imaging of the grating being shifted in the direction perpendicular to the grating lines, which aligns with our theoretical derivation. When we extract one row of data for spectral reconstruction, we find that the self-imaging and negative self-imaging of the grating overlap, causing a change in the period, as shown in Figure 4(b2). This results in errors in the spectral reconstruction. Therefore, during the alignment, it is essential to strictly ensure that the collimated light is perpendicular to the grating. From Figure 4(a3), when a spherical wave is normally incident on the grating, the period of the grating is magnified. When we extract one row of data, we notice a change in the contrast of the fringes. This is likely due to the low spatial coherence of the spherical wave, but the period of the Talbot fringes does not change, so it does not affect the spectral reconstruction.

**Figure 5:**
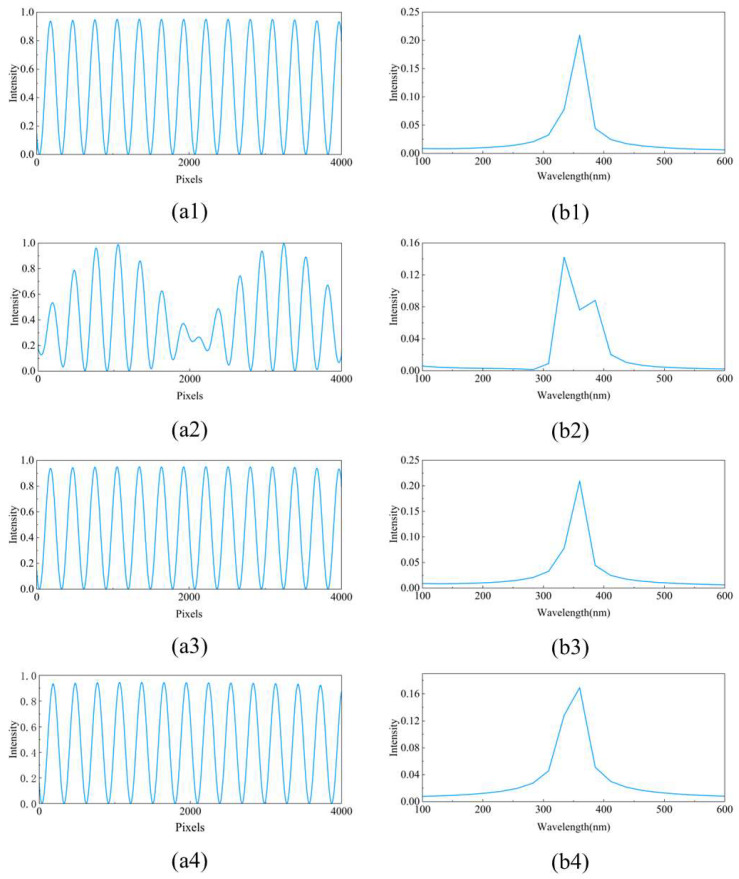
Single-row Talbot fringe and retrieved spectrum of the condition which (**a1**,**b1**) parallel light normal incident, (**a2**,**b2**) parallel light obliquely incident perpendicular to the grating line direction, (**a3**,**b3**) parallel light obliquely incident along the grating line direction, and (**a4**,**b4**) spherical light normal incident.

Next, we analyze the impact of different incident conditions on spectral reconstruction. For the case of oblique incidence of parallel light, a row of data at the center of the detector is used for spectral reconstruction. For the case of normal incidence of spherical waves, the distance between the point light source and the grating is set to 100 mm, and again, a row of data at the center of the detector is used for spectral reconstruction. The original data and retrieved spectrum for each of these conditions are shown in Figure 5.

From Figure 5, it can be seen that when parallel light is normally incident, when parallel light is obliquely incident along the grating lines, and when spherical waves are normally incident, the spectral reconstruction is unaffected by extracting the central row of data. However, when parallel light is obliquely incident along the direction perpendicular to the grating lines, as shown in Figure 5(a2), the overlap between the grating’s self-imaging and negative self-imaging causes the period of the Talbot fringes to change. This results in the main peak in the retrieved spectrum splitting into two sub-peaks, which leads to significant errors. Therefore, it is urgently necessary to address the impact of oblique incidence of parallel light along the perpendicular direction to the grating lines on spectral reconstruction.

## 4. Method

**Figure 6:**
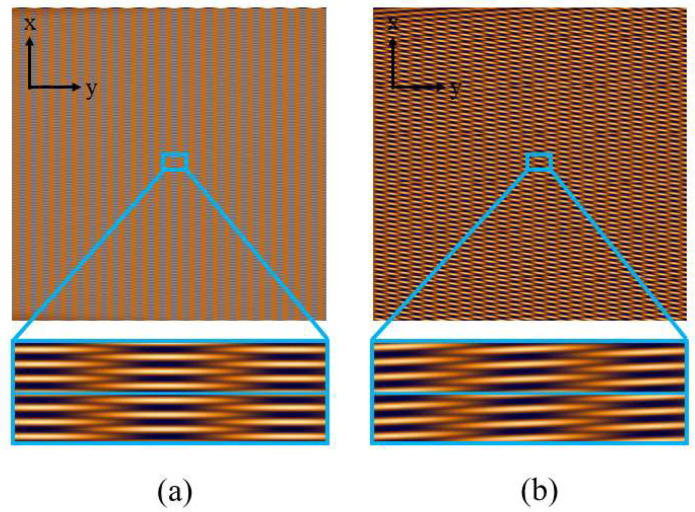
Talbot pattern at (**a**) normal incidence and (**b**) oblique incidence of parallel light.

From the discussion in the previous section, we found that the oblique incidence of parallel light along the direction perpendicular to the grating lines has a significant impact on spectral reconstruction. The main reason is that the self-imaging of the grating shifts along the direction perpendicular to the grating lines. Therefore, when we extract a row of data and perform Fourier transform, the self-imaging and negative self-imaging of the grating overlap, as shown in Figure 6, leading to errors in spectral reconstruction.

For ease of description, we define the x-direction as the periodic dimension and the y-direction as the spectral dimension. The stripes generated by periodic variations in the x-direction represent the grating period, while the stripes generated in the y-direction correspond to the self-imaging and negative self-imaging of the grating. We found that when capturing the Talbot carpet, in order to distinguish between the self-imaging and negative self-imaging of the grating, sampling needs to satisfy the Nyquist sampling theorem in the periodic dimension, meaning that the size of the detector pixels should be smaller than half the grating period. This requires the detector pixels to be very small, and due to the narrow width of the periodic dimension stripes, even slight tilting can cause overlap between self-imaging and negative self-imaging, leading to errors in spectral reconstruction. Inspired by the principle of Moiré fringes, we propose a method that involves placing a second grating with a different period in front of the detector to increase the width of the periodic dimension stripes, thus allowing for a larger tolerance for the tilt angle of parallel light along the direction perpendicular to the grating lines. Adding a second grating with a different period can introduce Moiré fringes in the periodic dimension. According to [19,20], when two gratings are placed at an angle *θ*, the period of the resulting Moiré fringes can be expressed as

(15) $$P=\frac{ab}{\sqrt{a^{2}+b^{2}-2ab\cos\theta }}$$

where *a* and *b* represent the periods of the two gratings, respectively. In this study, the two gratings are placed in parallel, meaning *θ* = 0, in which case,

(16) $$P=\frac{ab}{\left|a-b\right|}$$

**Figure 7:**
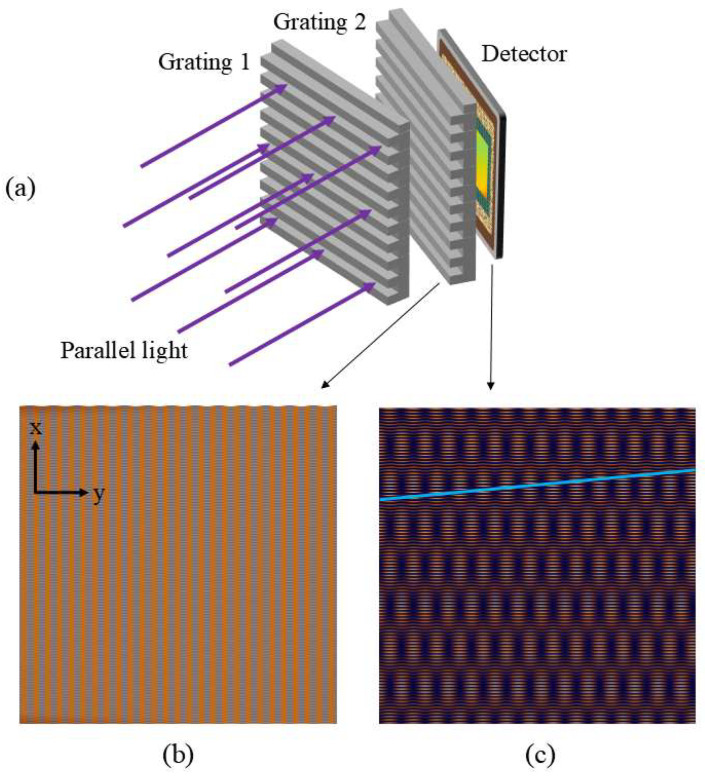
(**a**) Optical setup of this method, (**b**) Talbot pattern without the second grating, and (**c**) Talbot pattern with the second grating.

From the above equation, it can be observed that the smaller the difference between the periods of the two gratings, the larger the period of the Moiré fringes. Therefore, we aim to select two gratings with relatively small period differences. The specific implementation is shown in Figure 7.

When we do not add the second grating, the Talbot carpet captured by the detector is shown in Figure 7b, where the width of the periodic dimension stripes is very small, corresponding to the grating period. At this point, we add a second grating in front of the detector, ensuring that this grating is placed closely against the photosensitive surface of the detector and that its period differs from the first grating. When the incident light diffracts on the first grating, self-imaging occurs due to the Talbot effect, and the stripes generated by self-imaging will then be modulated in the periodic dimension by the second grating. Since the period of the second grating differs from that of the first, Moiré fringes will be generated in the periodic dimension without affecting the period in the spectral dimension. As a result, the width of the periodic dimension stripes increases, as shown in Figure 7c. Because the period in the periodic dimension becomes larger than that in the spectral dimension, the Nyquist sampling theorem shifts from the periodic dimension to the spectral dimension. At this point, the detector pixel size only needs to be smaller than half the Talbot fringe period. Due to the increased period of the periodic dimension stripes, even if the incident light undergoes a slight tilt in the direction perpendicular to the grating lines, the overlap of the fringes will not occur, thus not affecting the spectral reconstruction and significantly increasing the tolerance during alignment.

**Figure 8:**
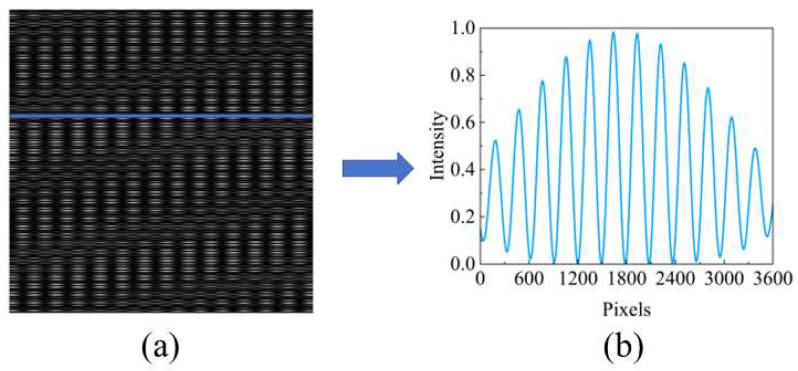
(**a**) Talbot carpet and (**b**) single-row Talbot fringe with oblique incidence of parallel light in the presence of a second grating.

Taking an incident angle of 0.2° with respect to the direction perpendicular to the grating lines as an example, when the second grating is not added, extracting a single row of data, as shown in Figure 8b, reveals that the self-imaging and negative self-imaging of the grating overlap in the central region. However, when the second grating is added and a single row of data is extracted, the increased width of the periodic dimension fringes prevents the overlap between the self-imaging and negative self-imaging of the grating, ensuring that spectral reconstruction remains unaffected. This demonstrates the effectiveness of our method.

## 5. Application

**Figure 9:**
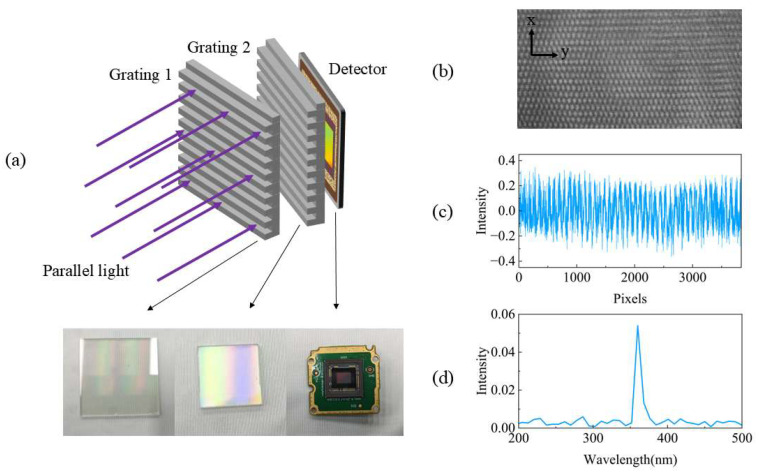
(**a**) Setup of the optical system, (**b**) Talbot carpet, (**c**) single-row Talbot fringe, and (**d**) reconstructed spectrum.

Based on this method, we constructed a Talbot wavemeter. A large sine-transmission grating with a grating period of 3.33 µm (300 lines/mm) and a size of 50 × 50 mm was used to generate the Talbot carpet. A smaller sine-transmission grating with a grating period of 3.28 µm (305 lines/mm) and a size of 20 × 20 mm was used to create Moiré fringes in the periodic dimension. A monochrome CMOS camera (Aptina MT9J003) was selected as the detector to capture the interference pattern, featuring a pixel pitch of 1.67 µm and a photosensitive area of 6.413 mm × 4.589 mm (pixels: 2748 × 3840). A UV-FN-360 nm laser (FWHM: 20 pm) combined with a BEW-5X beam expander was utilized as the light source to generate a collimated monochromatic laser beam. The experimental setup and corresponding spectral diagram are shown in Figure 9.

As shown in Figure 9b, due to the difference in line density between the second grating and the first grating, Moiré fringes are generated in the periodic dimension. As a result, the fringe width in the periodic dimension is greater than that in the spectral dimension. We extracted a central row of data and performed spectral retrieval, yielding the spectrum shown in Figure 9d. The resolution (FWHM) of the peak at 360 nm is 9 nm, which is consistent with the theoretical calculation [21].

For the Talbot wavemeter, in theory, as long as the detector is responsive, it can achieve full-wavelength measurement. Regarding the selection of grating periods, the first grating is used to generate Talbot fringes. While a smaller grating period leads to higher spectral resolution, it also results in narrower Talbot fringes. To ensure that the Talbot fringes can be effectively captured along the spectral dimension, a trade-off must be made between the detector pixel size and the Talbot fringe width, adhering to the Nyquist sampling criterion. Therefore, the optimal period of the first grating requires iterative testing during the engineering implementation of the wavemeter. Once the period of the first grating is determined, to generate longer moiré fringes in the periodic dimension, the second grating should have a period as close as possible to that of the first grating. Consequently, the choice of the second grating’s period is closely linked to that of the first grating. And at present, there are many methods that can be used for precision alignment, which can strictly ensure the parallel alignment of our front and back two gratings [22].

## 6. Conclusions

Variations in incident conditions can lead to errors in spectral reconstruction for the Talbot wavemeter. To address this issue, this paper first conducts a theoretical derivation based on Fresnel diffraction theory, analyzing the impact of different incident conditions on the Talbot interference pattern, which is further validated through simulations. Our findings indicate that oblique incidence of parallel light along the direction perpendicular to the grating lines has a significant effect on the interference pattern. To mitigate this issue, we propose adding a second grating with a different period in front of the detector to generate Moiré fringes in the periodic dimension, thereby increasing the tolerance for angular misalignment and resolving the problem. Finally, based on this method, we constructed a Talbot wavemeter, achieving a spectral resolution of 9 nm at 360 nm. This Talbot wavemeter is insensitive to temperature and air pressure, features a simple structure, and has a low cost. In scenarios requiring portable and cost-effective precise wavelength measurement, the Talbot wavemeter is the optimal choice, such as for spectral measurements in field or mobile environments.

## Data Availability

Data are contained within the article.
